# CaaX-Like Protease of Cyanobacterial Origin Is Required for Complex Plastid Biogenesis in Malaria Parasites

**DOI:** 10.1128/mBio.01492-20

**Published:** 2020-10-06

**Authors:** Thomas R. Meister, Yong Tang, Michael J. Pulkoski-Gross, Ellen Yeh

**Affiliations:** a Department of Molecular and Cellular Physiology, Stanford School of Medicine, Stanford, California, USA; b Department of Biochemistry, Stanford School of Medicine, Stanford, California, USA; c Department of Pathology, Stanford School of Medicine, Stanford, California, USA; d Department of Microbiology and Immunology, Stanford School of Medicine, Stanford, California, USA; e Chan Zuckerberg Biohub, San Francisco, California, USA; University of California Los Angeles

**Keywords:** *Plasmodium*, apicoplast, apicoplast biogenesis, CaaX protease, postprenylation processing, AMR4, ICMT, evolutionary biology, malaria, organelle biogenesis

## Abstract

*Plasmodium* parasites and related apicomplexans contain an essential “complex plastid” organelle of secondary endosymbiotic origin, the apicoplast. Biogenesis of this complex plastid poses a unique challenge requiring evolution of new cellular machinery. We previously conducted a mutagenesis screen for essential apicoplast biogenesis genes to discover organellar pathways with evolutionary and biomedical significance. Here we validate and characterize a gene candidate from our screen, Pf3D7_0913500. Using a conditional knockdown strain, we show that Pf3D7_0913500 depletion causes growth inhibition that is rescued by the sole essential product of the apicoplast, isopentenyl pyrophosphate (IPP), and results in apicoplast loss. Because Pf3D7_0913500 had no previous functional annotation, we name it apicoplast-minus IPP-rescued 4 (AMR4). AMR4 has an annotated CaaX protease and bacteriocin processing (CPBP) domain, which in eukaryotes typically indicates a role in CaaX postprenylation processing. Indeed, AMR4 is the only putative CaaX-like protease in *Plasmodium* parasites which are known to require protein prenylation, and we confirm that the conserved catalytic residue of AMR4 (E352) is required for its apicoplast function. However, we unexpectedly find that AMR4 does not act in a CaaX postprenylation processing pathway in Plasmodium falciparum. Instead, we find that AMR4 is imported into the apicoplast and is derived from a cyanobacterial CPBP gene which was retained through both primary and secondary endosymbiosis. Our findings suggest that AMR4 is not a true CaaX protease, but instead it performs a conserved, uncharacterized chloroplast function that has been retained for complex plastid biogenesis.

## Abstract (precis)

*Plasmodium* parasites, which cause malaria, and related apicomplexans are important human and veterinary pathogens. These parasites represent a highly divergent and understudied branch of eukaryotes, and as such often defy the expectations set by model organisms. One striking example of unique apicomplexan biology is the apicoplast, an essential but nonphotosynthetic plastid derived from an unusual secondary (eukaryote-eukaryote) endosymbiosis. Endosymbioses are a major driver of cellular innovation, and apicoplast biogenesis pathways represent a hot spot for molecular evolution. We previously conducted an unbiased screen for apicoplast biogenesis genes in P. falciparum to uncover these essential and innovative pathways. Here, we validate a novel gene candidate from our screen and show that its role in apicoplast biogenesis does not match its functional annotation predicted by model eukaryotes. Our findings suggest that an uncharacterized chloroplast maintenance pathway has been reused for complex plastid biogenesis in this divergent branch of pathogens.

## INTRODUCTION

*Plasmodium* spp., which cause malaria, and related apicomplexans are important human and veterinary pathogens. Beyond their biomedical significance, these parasitic protozoa represent a highly divergent and understudied branch of the eukaryotic tree, distinct from the well-studied model organisms in the opisthokont clade (e.g., yeast, mammals). As such, apicomplexans often defy the expectations of model eukaryotic biology, revealing surprising innovations that both highlight the diversity of eukaryotic life and can be leveraged for therapeutic intervention. One striking illustration of this unique biology is the nonphotosynthetic apicomplexan plastid, or apicoplast (1). The apicoplast is an example of a “complex plastid” derived from secondary endosymbiosis, in which an alga bearing a primary chloroplast was itself engulfed by another eukaryote (2). Although the apicoplast is no longer photosynthetic, it retains several metabolic pathways, is essential for parasite survival during human infection, and is a proven target of antiparasitic drugs (3–5).

Despite its importance to pathogenesis, little is known about how the apicoplast is maintained and replicated during the *Plasmodium* life cycle. Like other endosymbiotic organelles, the apicoplast cannot be formed *de novo* and must be faithfully inherited by its growth, division, and segregation into daughter parasites. The few details we know about these apicoplast biogenesis pathways reveal surprising innovations in eukaryotic evolution, particularly in membrane biology (6). Chloroplasts and endomembranes became uniquely intertwined during secondary endosymbiosis, which enclosed a double-membraned chloroplast in two outer membranes derived from the cell membrane of the algal symbiont and the phagocytic membrane of the engulfing eukaryote. Altogether, the apicoplast is surrounded by four membranes and is, in essence, an endosymbiotic organelle residing within a new membrane compartment. The transit of nucleus-encoded proteins to the apicoplast requires vesicle trafficking from the endoplasmic reticulum (ER) and a unique symbiont-derived ER-associated degradation-like machinery (SELMA) to cross the two outer membranes (7–10). Additionally, several members of the highly conserved eukaryotic autophagy pathway are unexpectedly required for apicoplast inheritance (11–14). While the repurposed SELMA and autophagy components hint at an abundance of molecular innovation in apicoplast biogenesis pathways, most of these pathways remain undiscovered.

To discover new genes required for apicoplast biogenesis and uncover more instances of molecular innovation, we previously conducted a mutagenesis screen to identify mutations that cause apicoplast loss (14). From this screen, we identified a mutation in a gene encoding a CaaX protease and bacteriocin processing (CPBP) family protein (Pf3D7_0913500, S347G). Known eukaryotic CPBP proteins that have been functionally characterized are CaaX proteases in the postprenylation processing pathway. During protein prenylation, a hydrophobic prenyl group is covalently attached to the cysteine residue of a C-terminal CaaX motif (where a is an aliphatic and X is any amino acid). Further modification of the prenylated CaaX by the postprenylation processing pathway includes cleavage of the terminal -aaX by a CaaX protease and carboxyl methylation of the neo C terminus by an isoprenylcysteine carboxyl methyltransferase (ICMT) on the ER membrane. Together, these posttranslational modifications mediate membrane association and regulate the function of many proteins (15, 16).

Protein prenylation is essential in Plasmodium falciparum (17, 18), suggesting the presence of a postprenylation processing pathway. It seemed likely that Pf3D7_0913500 would function as a CaaX protease in this pathway since it is the only P. falciparum gene with an annotation consistent with this function. The identification of Pf3D7_0913500 in our apicoplast biogenesis screen prompted several questions. (i) Is Pf3D7_0913500 essential specifically for apicoplast biogenesis? (ii) Does Pf3D7_0913500 function as a CaaX protease? (iii) Is CaaX postprenylation processing an apicoplast biogenesis pathway? To answer these questions, we utilized both reverse genetics and molecular evolutionary comparisons to probe the cellular role of Pf3D7_0913500.

## RESULTS

### AMR4 is essential and required specifically for apicoplast biogenesis.

**FIG 1:**
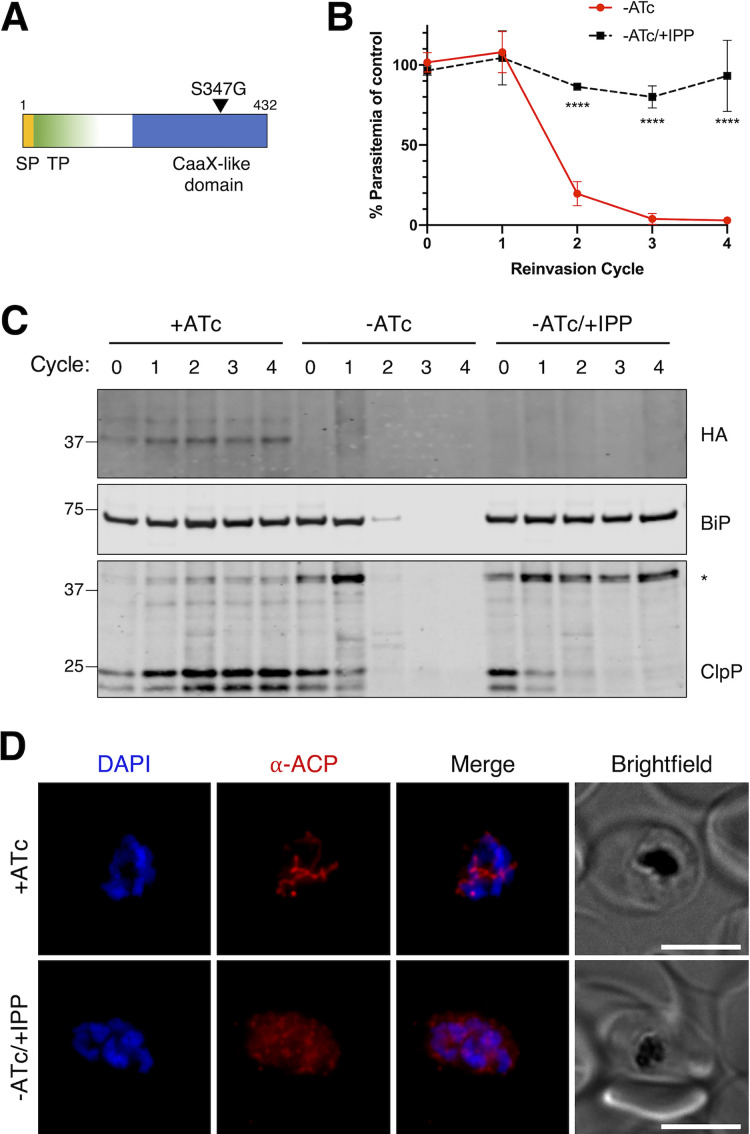
AMR4 is an essential protein required for apicoplast biogenesis. (A) Domain organization of AMR4 (Pf3D7_0913500) showing predicted signal peptide (SP), apicoplast transit peptide (TP), and the identified mutation. (B) Growth time course of AMR4 TetR-DOZI knockdown parasites in the absence of ATc (–ATc) with and without IPP. Data are normalized to the control with ATc (+ATc) at each time point. Error bars represent standard deviations of the means for 3 biological replicates. ****, *P* < 0.0001 compared to –ATc condition by repeated measures two-way ANOVA with Tukey’s multiple comparison test. (C) Western blot from growth time course of AMR4 knockdown parasites showing expression of AMR4-3xHA and processing of ClpP. Full-length ClpP (marked with an asterisk) is ∼40 kDa, while processed ClpP after removal of its transit peptide is ∼25 kDa. BiP serves as a loading control and marker for parasite growth. Blots from biological replicates are shown in Fig. S2A in the supplemental material. (D) Representative immunofluorescence images showing localization of the apicoplast marker ACP in +ATc and –ATc/+IPP parasites at reinvasion cycle 4 (α-ACP, anti-ACP antibody). Bars, 5 μm.

We previously performed a mutagenesis screen to identify new apicoplast biogenesis genes (14). Loss-of-function mutations in these genes disrupt inheritance of the apicoplast during parasite replication, resulting in organelle loss in daughter cells. Fifty-one mutants showing apicoplast loss were isolated and analyzed by whole-genome sequencing. To complete our analysis, we sequenced six additional mutant clones from this screen. Two of these clones contained previously identified mutations. We also report missense mutations in two new gene candidates, Pf3D7_1313400 and Pf3D7_1024900 (see Table S1 in the supplemental material). Altogether, out of 57 mutant clones analyzed, we identified 14 gene candidates. In our initial report, we validated three new apicoplast biogenesis genes that contained nonsense mutations. To continue validation of gene candidates identified by this screen, we next focused on genes containing missense mutations. Pf3D7_0913500 was identified by a mutation resulting in an S347G variant but has no known function in apicoplast biogenesis. However, it is predicted to be essential in blood-stage P. falciparum (19) and to contain an apicoplast targeting sequence (20, 21) (Fig. 1A), suggesting that it is an essential apicoplast protein consistent with a role in the organelle’s biogenesis.

10.1128/mBio.01492-20.6 TABLE S1 Raw nucleotide variants identified in sequenced clones. Download Table S1, XLSX file, 0.01 MB. Copyright © 2020 Meister et al. 2020 Meister et al. This content is distributed under the terms of the Creative Commons Attribution 4.0 International license.

To test the essentiality of Pf3D7_0913500, we generated a conditional knockdown strain by modifying the endogenous locus to include a C-terminal triple hemagglutinin (HA) tag and a 3′ untranslated region (UTR) RNA aptamer sequence that binds a tetracycline repressor (TetR) and development of zygote inhibited (DOZI) fusion protein (22, 23) (see Fig. S1A and B in the supplemental material). In the presence of anhydrotetracycline (ATc), the 3′ UTR aptamer is unbound and Pf3D7_0913500 is expressed. Removal of ATc from the media early in the parasite replication cycle resulted in efficient knockdown of Pf3D7_0913500 within the same cycle (Fig. 1C and Fig. S2A). We monitored parasite growth over several reinvasion cycles and found that depletion of Pf3D7_0913500 caused significant growth inhibition (Fig. 1B), confirming its essentiality. The only essential product of the apicoplast in blood-stage *Plasmodium* is isopentenyl pyrophosphate (IPP), such that supplementation with exogenous IPP rescues parasites with apicoplast defects, including complete loss of the organelle (24). Consistent with Pf3D7_0913500 having a specific apicoplast function, growth inhibition caused by Pf3D7_0913500 knockdown was fully reversed with the addition of IPP (Fig. 1B).

10.1128/mBio.01492-20.1 FIG S1 Genetic modification of endogenous AMR4 and *Pf*ICMT loci. (A) Schematic of CRISPR-Cas9-based genetic integration strategy used to generate conditional knockdown and knock-in mutation P. falciparum strains. (B) PCR products showing integrated or unintegrated AMR4 and *Pf*ICMT loci in parental (NF54^Cas9+T7 Polymerase^) or genome-edited parasites. (C) PCR products showing integrated or unintegrated AMR4 locus from knock-in mutations performed with IPP supplementation. (D) Sanger sequencing of integrated AMR4 locus from knock-in mutations performed with IPP supplementation. Download FIG S1, TIF file, 2.7 MB. Copyright © 2020 Meister et al. 2020 Meister et al. This content is distributed under the terms of the Creative Commons Attribution 4.0 International license.

10.1128/mBio.01492-20.2 FIG S2 Individual replicates of AMR4 and *Pf*ICMT Western blots. (A and B) Western blots from replicates of growth time courses of AMR4-3xHA (A) and *Pf*ICMT-3xHA (B) knockdown parasites showing protein expression levels and processing of ClpP. Full-length ClpP (marked with an asterisk) is ∼40 kDa, while processed ClpP after removal of its transit peptide is ∼25 kDa. BiP serves as a loading control and marker for parasite growth. (C and D) Western blots from replicates of transit peptide cleavage assays showing processing of AMR4-3xHA (C), *Pf*ICMT-3xHA (D), and ClpP (C and D) after 3 days of either no treatment or +10 μM actinonin/200 μM IPP. BiP serves as a loading control. (E) Western blots from replicates of protease protection assay. Organellar fraction from AMR4-3xHA parasites was treated with or without proteinase K in the presence or absence of Triton X-100. ClpR and ClpP serve as controls for internal apicoplast proteins, while ERD2 serves as a control for cytosol-exposed membrane proteins. Download FIG S2, TIF file, 2.6 MB. Copyright © 2020 Meister et al. 2020 Meister et al. This content is distributed under the terms of the Creative Commons Attribution 4.0 International license.

Essential apicoplast functions fall into two broad categories: those involved in organelle biogenesis and those involved solely in IPP production. As indicated, disruption of genes required for organelle biogenesis leads to apicoplast loss; in contrast, disruption of genes involved only in IPP production does not (25). To distinguish whether Pf3D7_0913500 knockdown caused apicoplast loss, we monitored apicoplast structure by immunofluorescence. During knockdown in IPP-rescued parasites, the apicoplast marker acyl-carrier protein (ACP) redistributed from a single organellar structure to diffuse puncta (Fig. 1D) as previously described for apicoplast loss. We additionally monitored apicoplast protein import as a readout for organelle loss. Most apicoplast proteins have an N-terminal transit peptide that is cleaved upon apicoplast import, and this processing event is eliminated in apicoplast-minus parasites (24). During Pf3D7_0913500 knockdown in IPP-rescued parasites, we observed the accumulation of the apicoplast protein caseinolytic protease P (ClpP) at its unprocessed molecular weight (Fig. 1C and Fig. S2A), indicating a disruption in apicoplast biogenesis processes (26). Together, these results confirm that Pf3D7_0913500 is specifically essential for an apicoplast biogenesis function. Because the gene had no previous functional annotation, we name it apicoplast-minus IPP-rescued 4 (AMR4), consistent with the loss-of-function phenotype observed in previously validated candidates from our screen.

### A conserved catalytic residue in the protease domain of AMR4 is required for its apicoplast function.

**FIG 2:**
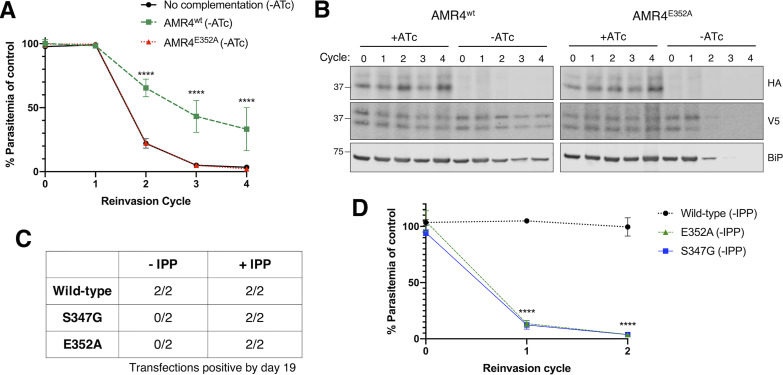
A conserved catalytic residue in the protease domain of AMR4 is required for its apicoplast function. (A) Growth time course of AMR4 TetR-DOZI knockdown parasites in the absence of ATc, complemented with either AMR4^wt^, AMR4^E352A^, or no complementation construct. Data are normalized to +ATc controls at each time point. Error bars represent standard deviations of the means for three biological replicates. ****, *P* < 0.0001 compared to no complementation control, repeated measures two-way ANOVA with Tukey’s multiple comparison test. (B) Western blot from growth time course of AMR4-3xHA knockdown parasites complemented with V5-tagged AMR4^wt^ or AMR4^E352A^. BiP serves as a loading control and marker for parasite growth. (C) Recovery of transfectants from endogenous AMR4 knock-in of wild-type, S347G, or E352A alleles. Transfections were performed with and without IPP in two independent experiments, and growth was monitored by luciferase signal. (D) Growth time course after removal of IPP from AMR4 knock-in strains recovered under IPP supplementation. Data are normalized to +IPP control at each time point. Error bars represent standard deviations of the means for two biological replicates. ****, *P* < 0.0001 compared to wild-type control by repeated measures two-way ANOVA with Tukey’s multiple comparison test.

The AMR4 mutation identified in our screen (S347G) is near the active site of the gene’s only annotated domain, a CaaX protease and bacteriocin processing (CPBP) domain. Mutagenesis and structural studies of CPBP domains indicate a catalytic mechanism in which a conserved glutamate activates a nucleophilic water molecule for proteolysis (27–30). Mutation of this catalytic glutamate was shown to completely abolish protease function without disrupting the stability or conformation of the protein (29). The catalytic glutamate is conserved in AMR4 at position 352. To test whether E352 is required for AMR4 function, we complemented the AMR4 knockdown strain with an episome expressing either wild-type (wild-type AMR4 [AMR4^wt^]) or protease-dead (AMR4 with E352 mutation [AMR4^E352A^]) constructs (31). Upon downregulation of endogenous AMR4 expression, we found that episomal expression of AMR4^wt^ partially but significantly complements the knockdown (Fig. 2A and B and Fig. S3). In contrast, episomal expression of AMR4^E352A^ shows no rescue, suggesting that the E352 residue is required for AMR4 function.

10.1128/mBio.01492-20.3 FIG S3 Episomally expressed AMR4^wt^ and AMR4^E352A^ are imported into the apicoplast. Western blot showing expression and processing of V5-tagged AMR4^wt^ and AMR4^E352A^ after 3 days of either no treatment or +10 μM actinonin/200 μM IPP to eliminate the apicoplast. AMR4^wt^ and AMR4^E352A^ are both properly imported into the apicoplast, as demonstrated by their apicoplast-dependent transit peptide cleavage. ClpP serves as a positive control for apicoplast import, and BiP serves as a loading control. The parental strain is AMR4 TetR-DOZI without episomal complementation. Download FIG S3, TIF file, 2.8 MB. Copyright © 2020 Meister et al. 2020 Meister et al. This content is distributed under the terms of the Creative Commons Attribution 4.0 International license.

It is likely that the partial functional complementation by AMR4^wt^ is due to altered abundance or stage-dependent expression from the nonnative promoter used for episomal expression. As an alternative to episomal expression, we directly introduced the E352A mutation or wild-type control into the endogenous AMR4 locus (Fig. S1A). We also introduced the S347G mutation identified in our screen to determine whether this mutation disrupted AMR4 function. These “knock-in” transfections were performed with and without IPP supplementation in two independent experiments. Wild-type transfectants were recovered from every transfection, while E352A and S347G transfectants were recovered only with IPP supplementation (Fig. 2C and Fig. S1C and D). For transfectants recovered with IPP supplementation, we then removed IPP from the media and tracked growth for two cycles. The E352A and S347G mutants had significantly reduced growth, indicating a dependence on exogenous IPP due to disruption of the apicoplast (Fig. 2D). In contrast, wild-type parasites grew similarly in the presence or absence of IPP supplementation, confirming that the IPP dependence of the E352A and S347G mutants was a result of the mutation and not prolonged growth with IPP supplementation. Together, these results show that the catalytic E352 residue in the CPBP domain is required for AMR4’s apicoplast function, indicating that AMR4 likely retains a protease activity. It additionally validates that the S347G mutation, which identified AMR4 in our initial screen, disrupts the protein’s function and caused the apicoplast loss phenotype in the original mutant.

The only known catalytic activity of eukaryotic CPBP domains is cleavage of a C-terminal CaaX motif (where a is an aliphatic and X is any amino acid) following cysteine prenylation as part of a postprenylation processing pathway (15, 16). Because CPBP catalysis is required for AMR4 function, we tested whether AMR4 has CaaX protease activity in yeast by functional complementation of a CaaX protease-knockout strain (32). Although AMR4 did not restore CaaX protease activity in the knockout yeast strain (Fig. S4), we were unable to detect tagged AMR4 to confirm protein expression and proper localization. Therefore, it remains unclear whether AMR4 retains CaaX protease activity.

10.1128/mBio.01492-20.4 FIG S4 AMR4 does not functionally complement CaaX proteases in yeast “halo” assay. Proper secretion of the yeast mating pheromone **a**-factor is dependent on CaaX prenylation and postprenylation processing. A mutant α-type strain (*MAT*α*^sst2^*) is growth arrested when exposed to secreted **a**-factor, such that when *MAT***a** cells are spotted over a lawn of *MAT*α*^sst^*^2^ cells, proper CaaX processing of **a**-factor results in a growth arrest “halo” around the spot (R. K. Chan, C. A. Otte, Mol Cell Biol 2:11−20, 1982, https://doi.org/10.1128/MCB.2.1.11). This assay has been used previously to test heterologous CaaX proteases and is sensitive enough to detect below 5% of wild-type **a**-factor processing (J. Cadiñanos, I. Varela, D. A. Mandel, W. K. Schmidt, et al., J Biol Chem 278:42091−42097, 2003, https://doi.org/10.1074/jbc.M306700200; J. Cadiñanos, W. K. Schmidt, A. Fueyo, I. Varela, et al., Biochem J 370:1047−1054, 2003, https://doi.org/10.1042/bj20021514; S. Michaelis, J. Barrowman, Microbiol Mol Biol Rev 76:626−651, 2012, https://doi.org/10.1128/MMBR.00010-12). We genetically modified *MAT***a** cells and used this halo assay as a read-out for functional CaaX protease activity. Double knockout of both yeast CaaX proteases, Rce1 and Afc1, abolished the halo phenotype as previously reported (C. E. Trueblood, V. L. Boyartchuk, E. A. Picologlou, D. Rozema, Mol Cell Biol 20:4381−4392, 2000,https://doi.org/10.1128/MCB.20.12.4381-4392.2000). Complementing this strain with *Sc*Rce1, which contains a CPBP domain, largely rescues the halo phenotype, indicating a restoration of CaaX postprenylation processing. We tested two AMR4 constructs, containing either the posttransit peptide protein (AMR4^112-432^) or the Rce1-like domain only (AMR4^197-432^), each fused with the N-terminal *Sc*Rce1 signal peptide for proper targeting. Both constructs failed to rescue the halo phenotype; however, we were unable to detect the tagged AMR4 constructs by Western blotting and therefore cannot confirm proper protein expression or localization. Images are representative of two independent experiments with two technical replicates per experiment. Download FIG S4, TIF file, 2.7 MB. Copyright © 2020 Meister et al. 2020 Meister et al. This content is distributed under the terms of the Creative Commons Attribution 4.0 International license.

### AMR4 is an imported apicoplast protein that does not share a postprenylation processing pathway with *Pf*ICMT.

**FIG 3:**
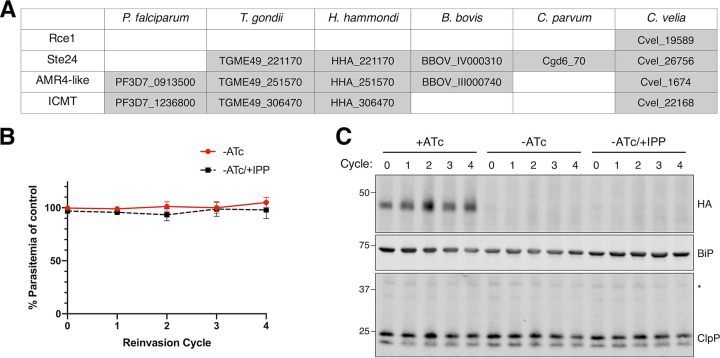
*Pf*ICMT is not an essential gene and does not have an apicoplast function. (A) Table showing the presence or absence of Rce1, Ste24, AMR4, and ICMT homologs in apicomplexans and chromerids. (B) Growth time course of *Pf*ICMT TetR-DOZI knockdown parasites in the absence of ATc with and without IPP. Data are normalized to +ATc control at each time point. Error bars represent standard deviations of the means for three biological replicates. No significant differences between means were detected at any time point by repeated measures two-way ANOVA with Tukey’s multiple comparison test. (C) Western blot from growth time course of *Pf*ICMT knockdown parasites showing expression of *Pf*ICMT-3xHA and processing of ClpP. Full-length ClpP (marked with an asterisk) is ∼40 kDa, while processed ClpP after removal of its transit peptide is ∼25 kDa. BiP serves as a loading control and marker for parasite growth. Blots from biological replicates are shown in Fig. S2B.

We also sought evidence that AMR4 might participate in a postprenylation processing pathway in *Plasmodium* parasites. Protein prenylation has been shown to be essential in P. falciparum (17, 18). It is presumed that *Plasmodium*, like other organisms, contains a two-step postprenylation processing pathway, in which a CaaX protease removes the terminal -aaX from prenylated proteins and the neo C terminus is then methylated by an isoprenylcysteine carboxyl methyltransferase (ICMT) (16). Two types of eukaryotic CaaX proteases are known: Rce1 glutamic proteases in the CPBP family and Ste24 zinc metalloproteases which are unrelated to the CPBP family. Unlike other apicomplexans and related chromerids, the only annotated CaaX-like protease in *Plasmodium* spp. is AMR4 (Fig. 3A). *Plasmodium* spp. also retain an ICMT homolog, suggesting that AMR4 and P. falciparum ICMT (*Pf*ICMT) may function together in a postprenylation processing pathway that is essential for apicoplast biogenesis.

To determine whether *Pf*ICMT is essential and required for apicoplast biogenesis, we generated a conditional knockdown strain using the TetR-DOZI system (22, 23) (Fig. S1A and B). As expected, removal of ATc caused a significant decrease in protein levels within a single replication cycle (Fig. 3C and Fig. S2B). However, *Pf*ICMT knockdown did not affect parasite growth over four replication cycles (Fig. 3B), indicating that it is nonessential in blood-stage culture. We also could not detect any defect in processing of the apicoplast protein ClpP (Fig. 3C and Fig. S2B), confirming that apicoplast biogenesis was not disrupted by *Pf*ICMT knockdown. The dispensability of *Pf*ICMT was surprising since it is proposed to act in an essential posttranslational modification pathway. It is possible that our knockdown was insufficient to completely disrupt *Pf*ICMT function; however, our result is also supported by an insertional transposon mutagenesis screen which assigns *Pf*ICMT as dispensable (19).

**FIG 4:**
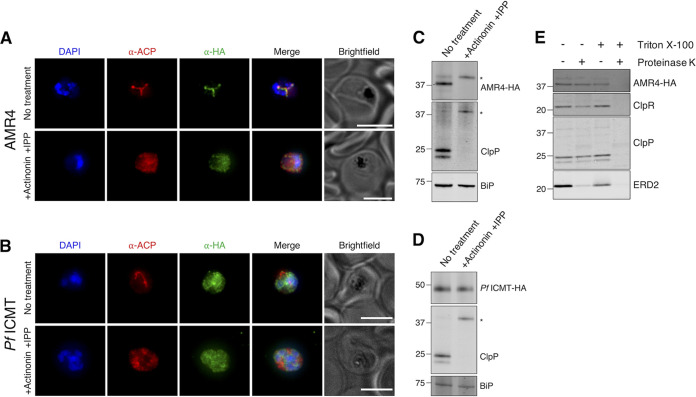
AMR4 is an imported apicoplast protein and does not share a postprenylation processing pathway with *Pf*ICMT. (A and B) Representative immunofluorescence images showing localization of AMR4-3xHA (A) and *Pf*ICMT-3xHA (B) compared to the apicoplast marker ACP after 3 days of either no treatment or treatment with 10 μM actinonin/200 μM IPP (+Actinonin +IPP). Bars, 5 μm. (C and D) Western blots showing processing of AMR4-3xHA (C), *Pf*ICMT-3xHA (D), and ClpP (C and D) after 3 days of either no treatment or +10 μM actinonin/200 μM IPP. BiP serves as a loading control. Blots from biological replicates are shown in Fig. S2C and D. (E) Western blot from protease protection assay. Organellar fraction from AMR4-3xHA parasites was treated with or without proteinase K in the presence or absence of Triton X-100. ClpR and ClpP serve as controls for internal apicoplast proteins, while ERD2 serves as a control for cytosol-exposed membrane proteins. Blots from biological replicates are shown in Fig. S2E.

The postprenylation processing enzymes typically colocalize to the ER membrane (33–35). We reasoned that if AMR4 and *Pf*ICMT perform postprenylation processing together, they should localize to the same membrane. To determine the cellular localization of AMR4 and *Pf*ICMT, we performed immunofluorescence and transit peptide cleavage assays with the endogenously tagged TetR-DOZI strains. Consistent with its predicted apicoplast targeting sequence, co-indirect immunofluorescence assay (co-IFA) shows that AMR4 colocalizes with the apicoplast marker ACP and is present on the organelle’s distinctive branching structures during schizogony (Fig. 4A). When parasites were treated with an inhibitor (actinonin) that causes apicoplast loss, ACP and AMR4 both redistribute to diffuse puncta, as previously observed for known apicoplast proteins (24). In contrast, *Pf*ICMT does not colocalize with ACP, and its localization is unaffected by actinonin treatment (Fig. 4B), indicating that it does not localize to the apicoplast.

We next assessed AMR4 and *Pf*ICMT protein cleavage as a marker for apicoplast import. Most apicoplast proteins have an N-terminal transit peptide that is cleaved upon apicoplast import, and this processing is eliminated in apicoplast-minus parasites (24). When the apicoplast is intact, AMR4 is detectable as a predominant mature band and a less abundant unprocessed band which likely indicates protein that is en route to the apicoplast. Upon apicoplast loss, AMR4 shifts exclusively to its unprocessed form (Fig. 4C and Fig. S2C), indicating that it is subject to apicoplast-dependent N-terminal cleavage. In contrast, *Pf*ICMT is detected as a single band which is unaffected by actinonin treatment (Fig. 4D and Fig. S2D), consistent with its nonapicoplast localization.

CaaX prenylation occurs in the cytoplasm, followed by postprenylation processing on a cytosolic membrane face. We reasoned that for AMR4 to perform a CaaX protease function, it should localize to the outer apicoplast membrane. To test this, we performed a protease protection assay in which we hypotonically lysed cell membranes of the endogenously tagged AMR4 strain while leaving organellar membranes intact. We then incubated this sample with proteinase K and assessed proteolysis by Western blotting. As expected, the mature forms of internal apicoplast proteins ClpP and ClpR are largely resistant to proteolysis (62.0% ± 10.9% and 48.2% ± 3.1%, mean ± standard deviation across three biological replicates). Similarly, the mature form of AMR4 is resistant to proteolysis (70.2% ± 3.8%), indicating that it is imported from the outer membrane of the apicoplast (Fig. 4E and Fig. S2E). In contrast, the cytosol-exposed ER/Golgi membrane protein ERD2 (36) is not resistant to proteolysis (6.2% ± 1.4%). When proteinase K is added in the presence of 1% Triton X-100 to disrupt organellar membranes, both cytosol-exposed and membrane-protected proteins are proteolyzed. We note that the integral membrane proteins ERD2 and AMR4 are also partially degraded after Triton X-100 lysis in the absence of proteinase K, likely indicating that the membrane-extracted proteins are exposed to endogenous proteases during incubation. On the basis of these results, we conclude that AMR4 is localized to the apicoplast and is imported into one of the inner membranes, unlike typical eukaryotic CaaX proteases. Additionally, *Pf*ICMT does not phenocopy or colocalize with AMR4, suggesting that they do not share a postprenylation processing pathway.

### AMR4 is derived from a prokaryotic CPBP gene acquired through endosymbiosis.

**FIG 5:**
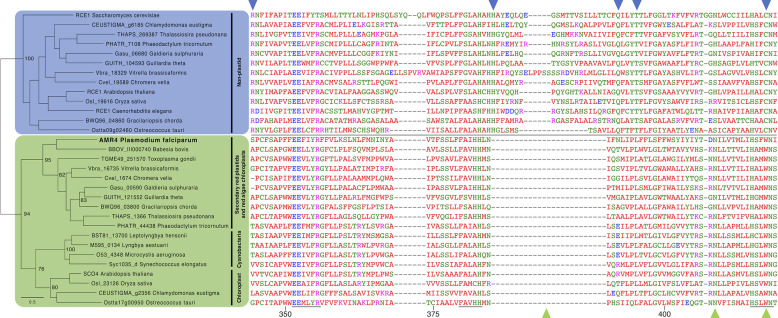
AMR4 is derived from a prokaryotic CPBP gene acquired through endosymbiosis. Phylogenetic analysis of selected CPBP proteins from cyanobacteria, primary and secondary plastids, and nonplastid Rce1s. Maximum likelihood phylogeny defines a monophyletic clade of cyanobacterial and chloroplast-targeted proteins along with AMR4 and its secondary plastid homologs. Outside of this clade are non-plastid-targeted Rce1 proteins from eukaryotes with and without plastids. Branch support values for well-supported major nodes are shown, out of 100 bootstrap intervals. Sequence alignment of the CPBP domain from each protein is shown. Arrowheads indicate residues that are conserved among nonplastid Rce1s (blue) or among cyanobacterial and plastid CPBPs (green). Amino acid numbering for *Pf*AMR4 is shown, and conserved CPBP motifs are underlined. Predicted targeting sequences for all proteins are shown in Table S2.

The CPBP family consists of two branches: eukaryotic Rce1 CaaX proteases and prokaryotic abortive infection (abi) proteases. Because AMR4 is apicoplast localized and does not function in a CaaX postprenylation processing pathway, we hypothesize that it may have originated from the prokaryotic chloroplast-like symbiont and not from a eukaryotic Rce1 from either the red algal symbiont or secondary host. To determine its origin, we generated a phylogeny of CPBP proteins from organisms along each step of these successive endosymbioses, including (i) cyanobacteria, (ii) eukaryotes without plastids, (iii) eukaryotes with primary chloroplasts, and (iv) eukaryotes with secondary red plastids. AMR4 is highly conserved in apicomplexans and related chromerids, with the exception of *Cryptosporidium* which has lost its apicoplast. The phylogeny shows with strong support that AMR4 and its secondary plastid homologs form a monophyletic clade with both cyanobacterial proteins and primary chloroplast proteins with predicted transit peptides, including Arabidopsis thaliana Sco4 (*At*Sco4) which is known to be chloroplast localized (37) (Fig. 5 and Table S2). Outside of this clade are eukaryotic Rce1 proteases (Rce1s) which do not have predicted chloroplast transit peptides, including confirmed ER CaaX proteases from yeast and *Arabidopsis* (33, 34). To prevent bias from different targeting signals in our analysis, we also generated a phylogeny using only the annotated CPBP domain (IPR003675) from each protein, which confirmed AMR4’s inclusion in the cyanobacterial/plastid clade (Fig. S5).

10.1128/mBio.01492-20.5 FIG S5 Phylogenetic analysis of CPBP domains confirms cyanobacterial origin of AMR4. Phylogenetic analysis of selected CPBP proteins from cyanobacteria, primary and secondary plastids, and nonplastid Rce1s. To prevent bias from different targeting sequences, only the annotated CPBP domain (IPR003675) from each protein was used. Maximum likelihood phylogeny defines a monophyletic clade of cyanobacterial and chloroplast-targeted proteins along with AMR4 and its secondary plastid homologs. Outside of this clade are non-plastid-targeted Rce1 proteins from eukaryotes with and without plastids. Branch support values for well-supported major nodes are shown, out of 100 bootstrap intervals. Download FIG S5, TIF file, 2.7 MB. Copyright © 2020 Meister et al. 2020 Meister et al. This content is distributed under the terms of the Creative Commons Attribution 4.0 International license.

10.1128/mBio.01492-20.7 TABLE S2 List of proteins and predicted localizations used for phylogenetic analysis. Download Table S2, XLSX file, 0.01 MB. Copyright © 2020 Meister et al. 2020 Meister et al. This content is distributed under the terms of the Creative Commons Attribution 4.0 International license.

Sequence alignment of these CPBP domains highlights several residues and motifs which are highly conserved within either eukaryotic Rce1s or cyanobacterium-derived proteins (Fig. 5). In each of these cases, AMR4 shares the conserved sequence with cyanobacterial proteins, but it does not share any of the Rce1-specific motifs. Taken together, these results confirm that AMR4 originated from a cyanobacterial CPBP gene which was retained through both primary and secondary endosymbiosis. This further suggests that AMR4 may share a molecular function with its homologs in chloroplasts and other complex plastids.

## DISCUSSION

As a result of its divergent evolution, thousands of genes in the P. falciparum genome remain unannotated (38). We previously designed an unbiased screen that allows for discovery of new genes with essential roles in apicoplast biogenesis (14). Here, we validate a previously uncharacterized gene candidate from our screen, AMR4, and show that it has an essential function in apicoplast biogenesis. AMR4 has an annotated CaaX protease and bacteriocin processing (CPBP) domain, which usually indicates a CaaX postprenylation processing function in eukaryotes. However, we provide three lines of evidence to demonstrate that AMR4 does not perform postprenylation processing. First, we show that the downstream postprenylation processing enzyme *Pf*ICMT does not phenocopy or colocalize with AMR4, suggesting that they do not share a pathway. Second, we show that AMR4 is imported into one of the inner apicoplast membranes. In contrast, protein prenylation and postprenylation processing typically occur on cytosolic membrane faces, and none of the P. falciparum prenyltransferases or known prenylated proteins are predicted to localize to the apicoplast (20, 21, 39, 40). Finally, we find that AMR4 did not evolve from a eukaryotic CaaX protease, but instead it is derived from a cyanobacterial CPBP gene which was retained through both primary and secondary endosymbiosis.

Although AMR4 is not a CaaX protease, we show that the conserved catalytic residue of its CPBP domain is required for its role in apicoplast biogenesis. This suggests that AMR4 has catalytic activity and is likely to be a protease, as it retains all three conserved CPBP active site motifs (Fig. 5). We have had preliminary success expressing and purifying recombinant AMR4; however, we have so far been unable to detect *in vitro* proteolytic activity against a diverse peptide library. Further work will be required to characterize the catalytic activity of AMR4.

Our finding that AMR4 was derived from a cyanobacterial CPBP gene, as opposed to a eukaryotic CaaX protease from either the red algal endosymbiont or secondary host, suggests that it might exist as part of a conserved chloroplast pathway. Indeed, a chloroplast-targeted CPBP protein in A. thaliana (*At*Sco4) was previously shown to play a role in chloroplast maintenance and light acclimation (37). It is possible that AMR4 and its secondary plastid homologs act in a similar pathway as *At*Sco4. However, many primary chloroplasts, including *A. thaliana* have an expanded repertoire of uncharacterized CPBP proteins (41), therefore limiting our ability to infer AMR4 function. Altogether, we consider it likely that AMR4 resides in one of the inner two apicoplast membranes and performs a conserved but unknown role in plastid maintenance. Future work will focus on identifying cellular substrates of AMR4 proteolysis and uncovering the apicoplast biogenesis pathway in which it functions.

Our findings also raise questions about the state of postprenylation processing in *Plasmodium*. Do *Plasmodium* spp. possess a functional CaaX protease? AMR4 is the only identifiable CaaX-like protease; however, our results show that it does not perform a CaaX function. Therefore, it appears that *Plasmodium* spp. might lack a CaaX protease, in contrast to other apicomplexan parasites which retain a Ste24 homolog that could perform this function (Fig. 3A). Alternatively, *Plasmodium* spp. may possess a cryptic CaaX protease which has not yet been identified, either a highly divergent Ste24 homolog or an unrelated protease which has evolved a postprenylation processing function.

If *Plasmodium* lacks a CaaX protease, is the postprenylation processing pathway being lost? Genome reduction is a major driving force during parasite evolution, and therefore, nonessential pathways are likely to be lost (42). While prenylation is known to be essential in P. falciparum (17, 18), we show that the postprenylation processing enzyme *Pf*ICMT is nonessential in blood-stage culture. This is consistent with results from previous whole-genome essentiality screens which assign “dispensable” annotations to *Pf*ICMT as well as the Toxoplasma gondii Ste24 and ICMT homologs (19, 43). Additionally, several apicomplexan genera, including *Babesia* and *Cryptosporidium* lack ICMT homologs (Fig. 3A), suggesting partial loss of the postprenylation processing pathway in related parasites. However, we cannot rule out the possibility that apicomplexan Ste24 or ICMT homologs are required in other parasite life stages and growth conditions or that cryptic ICMT activity may be required.

Do prenylated CaaX proteins undergo further modification in *Plasmodium*? P. falciparum has a reduced prenylome, containing only eight prenylated CaaX proteins (39, 40). If none of these proteins require postprenylation processing to function, then it is likely that the pathway would be lost. Indeed, previous work in model systems shows that not all prenylated CaaX proteins undergo further modification from postprenylation processing enzymes (44, 45). Similar proteomic approaches will be required in *Plasmodium* to determine whether any prenylated proteins undergo CaaX cleavage and/or carboxyl methylation, indicating whether the parasites retain a cryptic CaaX protease or whether the postprenylation processing pathway has been lost.

More broadly, our results highlight the need to study eukaryotes that diverge from model organisms. We provide another example of apicomplexan biology defying the expectations set by model eukaryotes. Our screen provides an unbiased method to identify divergent genes that act in essential apicoplast biogenesis pathways. Apicoplast biogenesis is a hot spot for molecular innovation, as demonstrated by examples of genes being reused (e.g., AMR4), repurposed (e.g., Atg8, SELMA, AMR1), or newly invented (e.g., AMR3) to maintain the complex plastid. In each of these cases, gene annotations based on model eukaryotes fail to accurately represent biological function. These novel pathways must be uncovered through biochemical, cellular, and comparative evolutionary approaches to better understand the true diversity of eukaryotic life.

## MATERIALS AND METHODS

### Ethics statement.

Human erythrocytes were purchased from the Stanford Blood Center (Stanford, CA) to support *in vitro* Plasmodium falciparum cultures. Because erythrocytes were collected from anonymized donors with no access to identifying information, institutional review board (IRB) approval was not required. All consent to participate in research was collected by the Stanford Blood Center.

### Parasite culture and transfections.

Plasmodium falciparum parasites were grown in human erythrocytes (Stanford Blood Center) at 2% hematocrit in RPMI 1640 medium (Gibco) supplemented with 0.25% Albumax II (Gibco), 2 g/liter sodium bicarbonate (Fisher), 0.1 mM hypoxanthine (Sigma), 25 mM HEPES, pH 7.4 (Sigma), and 50 μg/liter gentamicin (Gold Biotechnology) at 37°C, 5% O_2_, and 5% CO_2_.

Transfections were performed into NF54^Cas9+T7 Polymerase^ parasites (43) (a kind gift from Jacquin Niles) using variations of the spontaneous uptake method (23, 46). In the first variation, 100 μg of each plasmid was ethanol precipitated and resuspended in 0.2-cm electroporation cuvettes in 30 μl sterile Tris-EDTA (TE) buffer, 170 μl cytomix, and 200 μl packed erythrocytes. Erythrocytes were electroporated at 310 V, 950 μF, infinite resistance in a Gene Pulser Xcell electroporation system (Bio-Rad) before allowing parasites to invade. Drug selection was initiated 3 days after transfection. In the second variation, 50 μg of each plasmid was ethanol precipitated and resuspended in 0.2-cm electroporation cuvettes in 100 μl TE buffer, 100 μl RPMI 1640 containing 10 mM HEPES-NaOH (pH 7.4) and 200 μl packed erythrocytes. Erythrocytes were pulsed with eight square wave pulses of 365 V × 1 ms separated by 0.1 s. Erythrocytes were allowed to reseal for 1 h in a 37°C water bath before allowing parasites to invade. Drug selection was initiated 4 days after transfection.

All transfectants were selected with 2.5 μg/ml Blasticidin S (Research Products International) and maintained in the presence of 500 nM anhydrotetracycline (ATc) (Sigma). Additionally, strains carrying the pfYC110 episome were selected and maintained with 125 μg/ml G418 sulfate (Corning). Integration of plasmids into endogenous loci was confirmed by PCR. Additionally, integration of the correct allele from each knock-in mutation was confirmed by Sanger sequencing. Growth of knock-in transfections was monitored using the *Renilla* luciferase assay system (Promega) and GloMax 20/20 luminometer (Promega).

### Vector construction.

Oligonucleotides and gBlocks were ordered from IDT. Molecular cloning was performed using Gibson Assembly (NEB) or In-Fusion Cloning (Clontech). All primer and gBlock sequences are provided in Table S3 in the supplemental material.

10.1128/mBio.01492-20.8 TABLE S3 Primer and gBlock sequences used in this study. Download Table S3, XLSX file, 0.01 MB. Copyright © 2020 Meister et al. 2020 Meister et al. This content is distributed under the terms of the Creative Commons Attribution 4.0 International license.

For CRISPR-Cas9-based editing of endogenous Pf3D7_0913500 (AMR4) and Pf3D7_1236800 (*Pf*ICMT) loci, single guide RNAs (sgRNAs) were designed using the eukaryotic CRISPR guide RNA/DNA tool (http://grna.ctegd.uga.edu/). To generate a linear plasmid for CRISPR-Cas9-based editing, left and right homology regions were amplified for each gene, and a gBlock containing the recoded sequence C terminal of the CRISPR cut site and a triple hemagglutinin (HA) tag was synthesized with appropriate overhangs for Gibson Assembly. This fragment and the left homology region were simultaneously cloned into the FseI/ApaI sites of the linear plasmid pSN054-V5. Next, the appropriate right homology region and a gBlock containing the sgRNA expression cassette were simultaneously cloned into the AscI/I-SceI sites of the resultant vectors to generate each TetR-DOZI plasmid. Plasmids for knock-in mutations of the endogenous Pf3D7_0913500 locus were generated as described above, using C-terminal recoded gBlocks with the intended mutation.

To generate the episomal complementation constructs for Pf3D7_0913500, gBlocks containing the recodonized N terminus and C terminus (either wild type or E352A mutant) of Pf3D7_0913500 were stitched together by overlap extension PCR and cloned into the AvrII/SacII sites of the pfYC110 plasmid (31) using In-Fusion Cloning.

To generate the mating hormone A-factor 1 (MFA1) expression plasmid, the MFA1 5′ untranslated region (UTR), coding sequence, and 3′ UTR were amplified from Saccharomyces cerevisiae genomic DNA and cloned into the BamHI/HindIII sites of the pRS316 plasmid (32) using In-Fusion Cloning. To generate the S. cerevisiae Rce1 (*Sc*Rce1) complementation construct, the 5′ UTR and *Sc*Rce1 coding sequence were individually amplified. These fragments and a gBlock containing a C-terminal Flag tag and the *Sc*Rce1 3′ UTR were cloned into the SacII/HindIII sites of the pRS315 plasmid (32). To generate Pf3D7_0913500 complementation constructs with the *Sc*Rce1 signal peptide, amino acids 112 to 432 or 197 to 432 were amplified from a gBlock containing Pf3D7_0913500 recodonized for Escherichia coli expression. These fragments and gBlocks containing the *Sc*Rce1 signal peptide with appropriate overhangs were then cloned into the BamHI/PstI sites of the resultant *Sc*Rce1 complementation construct using In-Fusion Cloning.

### Western blotting.

Parasites were separated from erythrocytes by lysis in 0.1% saponin for 5 min on ice. Parasite pellets were washed twice with ice-cold phosphate-buffered saline (PBS), resuspended in PBS containing 1× NuPAGE lithium dodecyl sulfate (LDS) sample buffer with 50 mM dithiothreitol (DTT), and boiled for 10 min before separation on NuPAGE gels (Invitrogen). Gels were transferred onto nitrocellulose membranes using the Trans-Blot Turbo system (Bio-Rad). Membranes were blocked in 0.1% Hammarsten casein (Affymetrix) in 0.2× PBS with 0.01% sodium azide. Antibody incubations were performed in a 1:1 mixture of blocking buffer and Tris-buffered saline with Tween 20 (TBST; 10 mM Tris [pH 8.0], 150 mM NaCl, 0.25 mM EDTA, 0.05% Tween 20). Blots were incubated with primary antibody for either 1 h at room temperature or at 4°C overnight using the following dilutions: 1:1,000 mouse anti-HA 2.2.14 (Thermo Fisher catalog no. 26183), 1:4,000 rabbit anti-*Pf*ClpP and 1:4,000 rabbit anti-*Pf*ClpR (47) (kind gifts from Walid Houry), 1:20,000 mouse anti-Plasmodium yoelii BiP (anti-*Py*BiP) (a kind gift from Sebastian Mikolajczak and Stefan Kappe), 1:1,000 rabbit anti-ERD2 (BEI MRA-1), 1:1,000 rabbit anti-V5 (Cell Signaling catalog no. 13202). The blots were washed three times in TBST and were incubated for 1 h at room temperature in a 1:10,000 dilution of an appropriate fluorescent secondary antibody (Li-COR Biosciences). The blots were washed three times in TBST and once in PBS before imaging on a Li-COR Odyssey imager.

### Actinonin treatment and IPP rescue.

To generate apicoplast-minus parasites, ring-stage cultures were treated with 10 μM actinonin (Sigma catalog no. A6671) and 200 μM isopentenyl pyrophosphate (IPP) (Isoprenoids LLC) for 3 days.

### Protease protection assay.

Fifty milliliters of schizont-stage parasite culture at 2% hematocrit and ∼5% parasitemia was separated from erythrocytes by lysis in 0.1% saponin for 5 min on ice. Parasite pellets were washed 3 times with ice-cold PBS and lysed in hypotonic lysis buffer (20 mM HEPES-NaOH [pH 7.2]) by passing through a 27-gauge needle 30 times. Lysate was centrifuged in assay buffer (50 mM HEPES-NaOH [pH 7.4], 50 mM NaCl, 250 mM sucrose) three times for 10 min at 1,500 × *g* to remove unbroken cells and debris. The lysate was split into four samples: (i) no treatment, (ii) 50 μg/ml proteinase K (Thermo Fisher catalog no. EO0491), (iii) 1% Triton X-100 (Sigma catalog no. 45ZH27), and (iv) 50 μg/ml proteinase K and 1% Triton X-100. After incubating for 30 min on ice, proteolysis was stopped by addition of 1 mM phenylmethylsulfonyl fluoride (PMSF) (Sigma catalog no. P7626). Samples were analyzed by Western blotting as described above. Band intensities were quantified using Image Studio Lite software (Li-COR Biosciences).

### Knockdown growth assays.

Ring-stage TetR-DOZI strain parasites were washed twice in culture medium to remove ATc. The parasites were divided into three cultures supplemented with 500 nM ATc, no ATc, or no ATc plus 200 μM IPP. Samples were collected at the schizont stage of each growth cycle for flow cytometry analysis and Western blotting. Parasites in each condition were diluted equally into fresh medium with 2% hematocrit for four reinvasion cycles. For parasitemia measurements, aliquots of culture were incubated with 16.67 μM dihydroethidium (Thermo Fisher) for 15 min. Parasites were analyzed on a BD Accuri C6 flow cytometer, and 100,000 events were recorded for each condition. Two-way analyses of variances (ANOVAs) were performed in GraphPad Prism. For Western blots, equal volumes of culture were loaded for each treatment condition of a strain.

### Immunofluorescence microscopy.

Parasite cultures were washed once in PBS and were fixed with 4% paraformaldehyde (Electron Microscopy Sciences catalog no. 15710) and 0.0075% glutaraldehyde (Electron Microscopy Sciences catalog no. 16019) in PBS for 20 min. Cells were washed once in PBS and allowed to settle onto poly-l-lysine-coated coverslips (Corning) for 60 min. Coverslips were washed once with PBS, permeabilized in 0.1% Triton X-100 in PBS for 10 min, and washed twice more in PBS. Cells were treated with PBS containing 0.1 mg/ml for 10 min, washed once in PBS, and blocked in PBS containing 5% bovine serum albumin (BSA) (5% BSA/PBS). Coverslips were incubated overnight at 4°C in primary antibody diluted in 5% BSA/PBS using the following concentrations: 1:500 rabbit anti-*Pf*ACP (48) (kind gift from Sean Prigge) and 1:100 rat anti-HA 3F10 (Sigma catalog no. 11867423001). Coverslips were washed three times in PBS, incubated with secondary antibodies donkey anti-rabbit 568 (Thermo Fisher catalog no. A10042) and goat anti-rat 488 (Thermo Fisher A-11006) at 1:3,000 dilution, and washed three times in PBS. Coverslips were mounted onto slides with ProLong Gold antifade reagent with 4′,6′-diamidino-2-phenylindole (DAPI) (Thermo Fisher) and were sealed with nail polish prior to imaging.

Cells were imaged with 100×, 1.4 numerical aperture (NA) or 100×, 1.35 NA objectives on an Olympus IX70 microscope with a DeltaVision system (Applied Precision) controlled with SoftWorx version 4.1.0 and equipped with a CoolSnap-HQ charge-coupled-device (CCD) camera (Photometrics). Images were captured as a series of z-stacks separated by 0.2-μm intervals and displayed as maximum intensity projections. Brightness and contrast were adjusted using Fiji (ImageJ) for display purposes.

### Yeast transformation and halo assay.

Transformation was performed as described previously (49). MFA1 expression plasmid along with complementation constructs expressing *Sc*Rce1, amino acids 112 to 432 of AMR4 (AMR4^112-432^), amino acids 197 to 432 of AMR4 (AMR4^197-432^), or empty vector were transformed into either AFC1 RCE1 (JRY5460), *afc1*Δ RCE1 (JRY6095), AFC1 *rce1*Δ (JRY5462), or *afc1*Δ *rce1*Δ (JRY5463) S. cerevisiae strains (32).

For halo assays (32, 50), cultures of *Mat*α *sst2* (JRY3343) and transformed *MAT***a** cells were grown overnight. A total of 4 × 10^6^ *MAT*α *sst2* cells were spread onto prewarmed plates containing yeast extract-peptone-dextrose (YPD) plus 0.04% Triton X-100 and allowed to dry. *MAT***a** cells (1 × 10^6^ cells) in 5 μl were then spotted onto the lawn and allowed to dry. Plates were incubated at 30°C for 2 or 3 days before imaging. Image color was inverted, and brightness and contrast were adjusted in Fiji (ImageJ) for display purposes.

### Sequence analysis and phylogeny.

Protein sequences were retrieved from EuPathDB (51) and UniProt (52). The CPBP domain of each gene was annotated using InterProScan (53). Sequences were aligned using MAFFT (54), and unrooted maximum likelihood phylogenies were performed using IQ-TREE (55) with default settings and 100 standard bootstrap intervals. Phylogenetic trees were then imported into FigTree (v.1.4.4) and midpoint rooted. Targeting sequences for each protein were predicted using TargetP-2.0 (56) (Table S2). Percent identity of each protein to AMR4 was calculated using Clustal Omega (57) (Table S2).

### Whole-genome sequencing and SNP analysis.

Isolation of apicoplast-minus mutant clones and DNA extraction were described previously (14). Genomic DNA samples from six additional clones were used to generate paired-end libraries using the NEB Next Ultra FS II kit with eight PCR cycles. Libraries were sequenced on an Illumina NextSeq 500 using 2 × 150 bp paired-end sequencing (Chan Zuckerburg Biohub). Single nucleotide polymorphism (SNP) analysis was performed using a custom script as described previously (14). Briefly, adapters were removed using cutadapt, and sequencing reads were aligned to the P. falciparum 3D7 (version 35) genome using Bowtie2. PCR duplicates were removed, and raw SNPs (base quality ≥ 20) were called using Samtools. Bcftools was used to generate the raw variant list for parental (allele frequency ≥ 0.01, depth ≥ 1) and mutant (allele frequency ≥ 0.9, depth ≥ 20) strains. Variants were filtered to only include protein-coding mutations, and variants found in the parental strain were subtracted from the mutant variant list. Mutations in hypervariable gene families and mutations that were previously annotated in PlasmoDB were excluded from the final variant list (Table S1).

### Data availability.

Raw whole-genome sequencing data are available via the SRA repository (accession number PRJNA655924). SRA accession numbers for all samples are reported in Table S1.
