# Supplementary figures and images for: CaaX-Like Protease of Cyanobacterial Origin Is Required for Complex Plastid Biogenesis in Malaria Parasites
Source: mBio. 2020 Oct 6;11(5):e01492-20. doi: 10.1128/mBio.01492-20 (PMC7542359; doi:10.1128/mBio.01492-20)

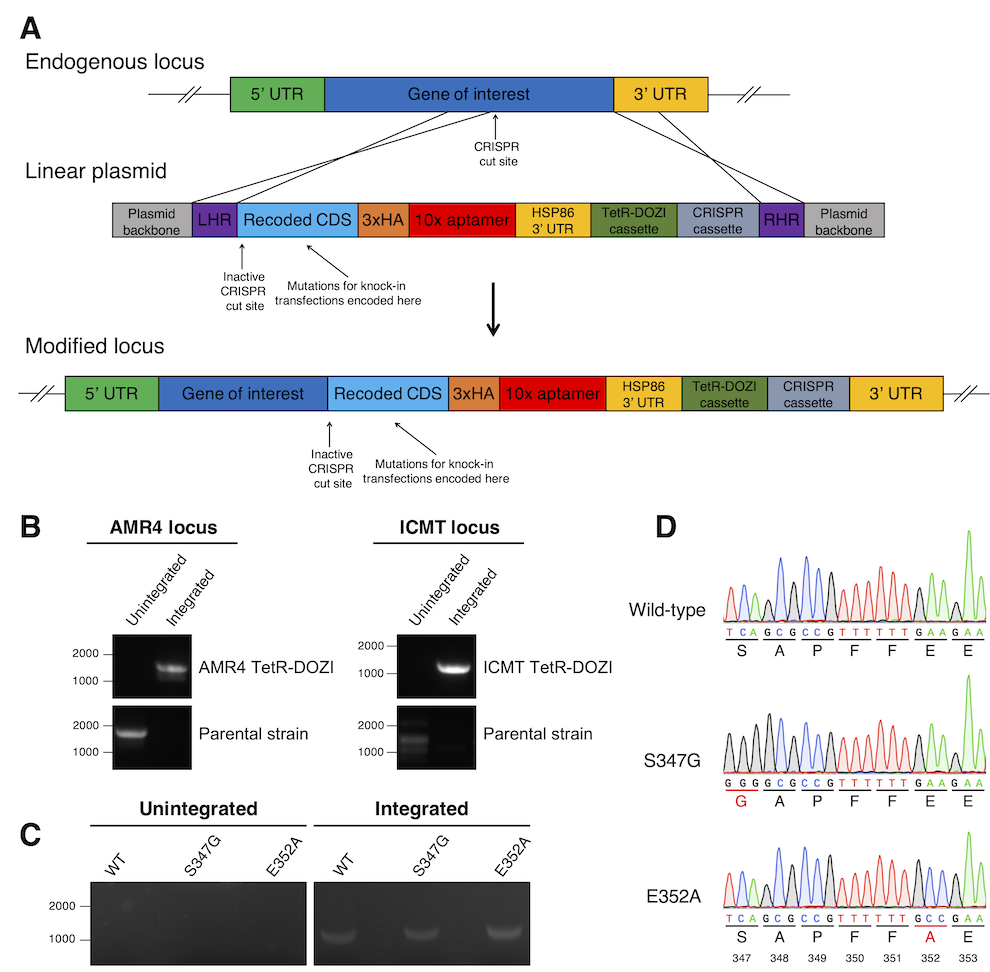

Supplement: FIG S1 [file mBio.01492-20-sf001.tif]

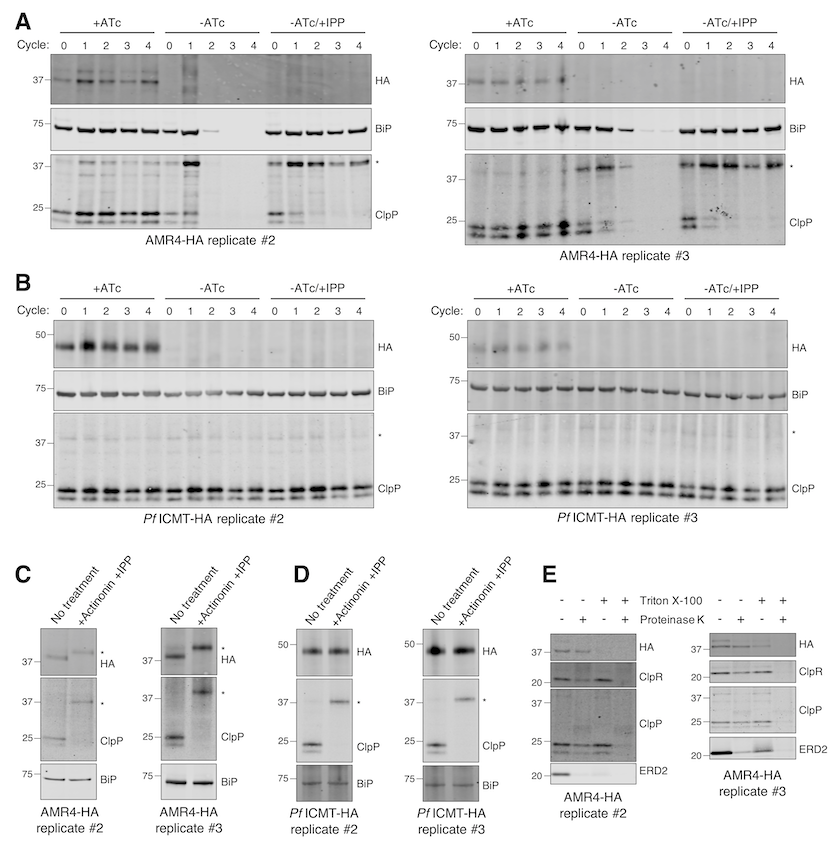

Supplement: FIG S2 [file mBio.01492-20-sf002.tif]

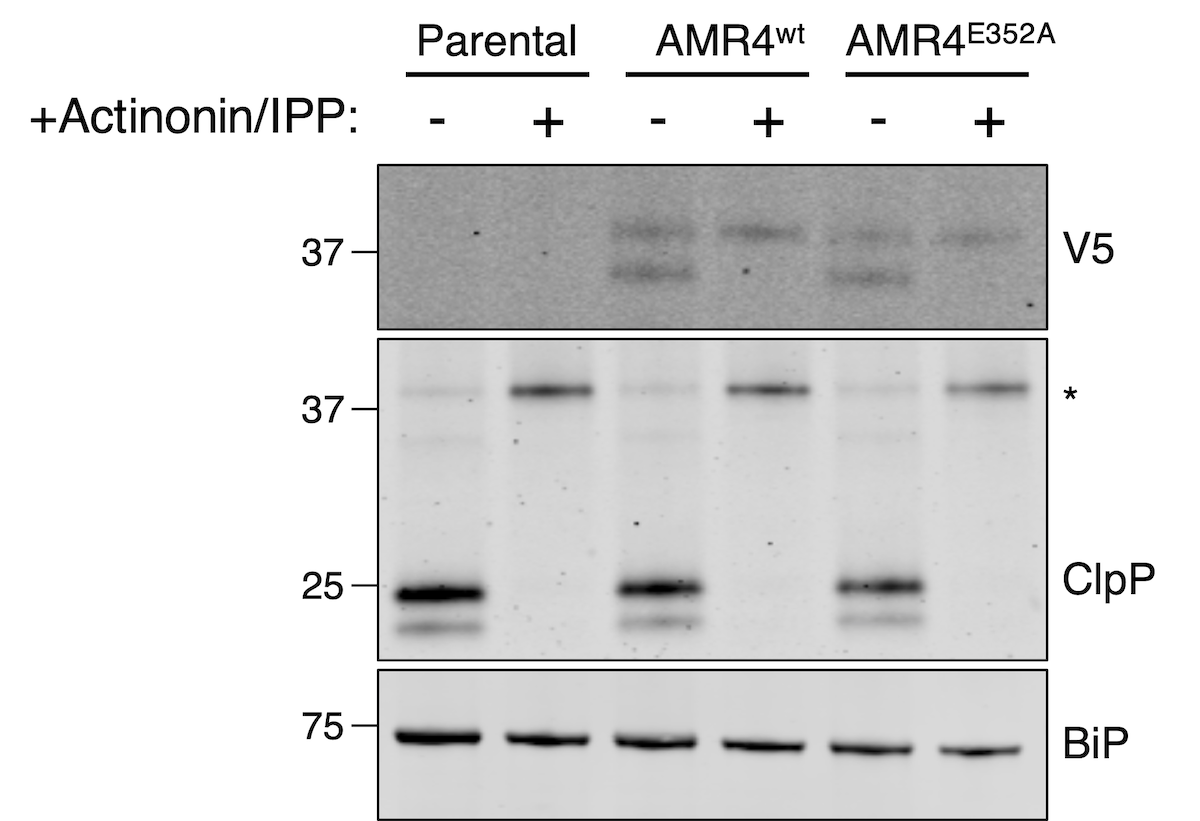

Supplement: FIG S3 [file mBio.01492-20-sf003.tif]

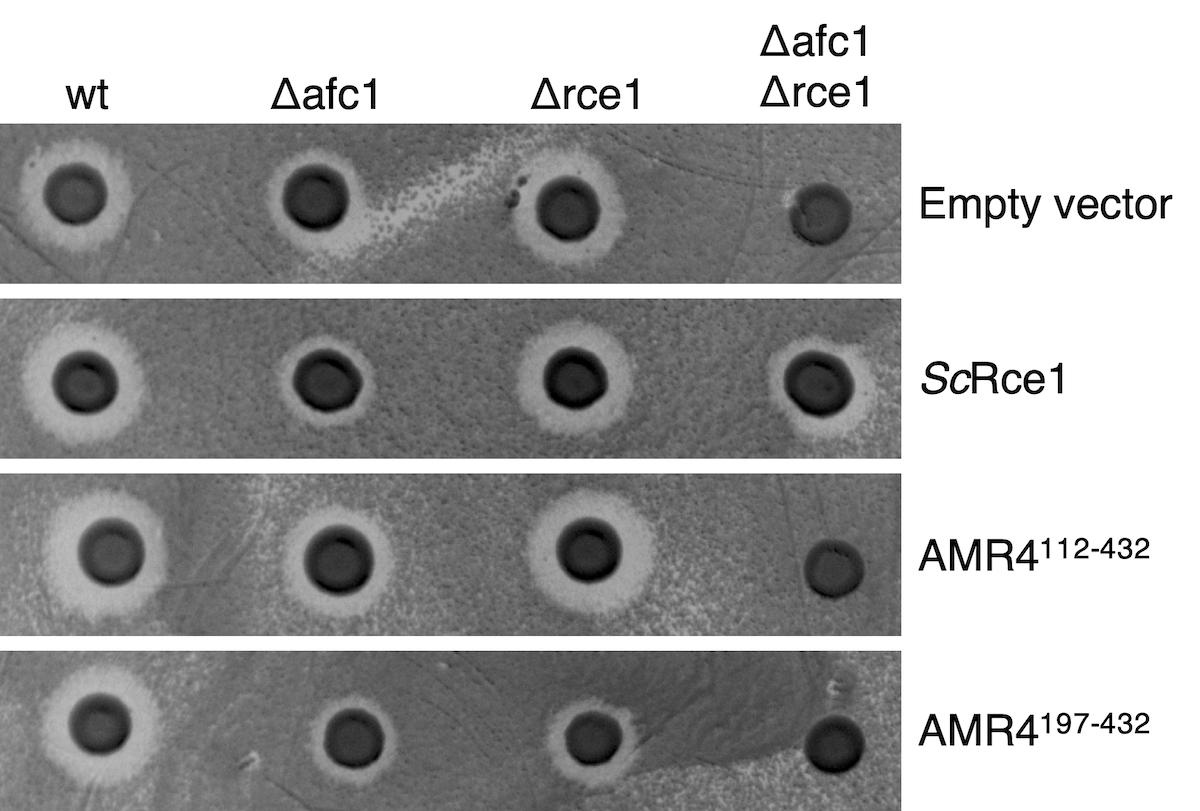

Supplement: FIG S4 [file mBio.01492-20-sf004.tif]

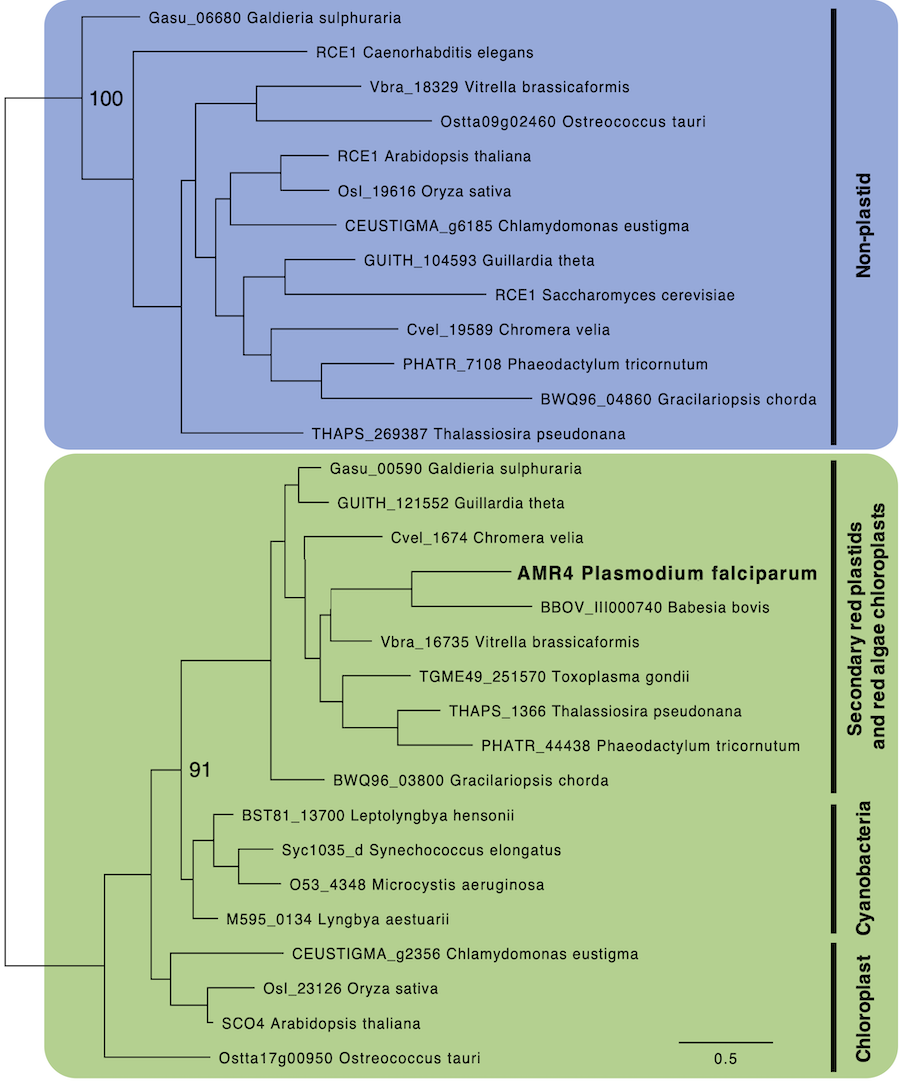

Supplement: FIG S5 [file mBio.01492-20-sf005.tif]
